# Supplementary material for: Complex Actions of FKBP12 on RyR1 Ion Channel Activity Consistent with Negative Co-Operativity in FKBP12 Binding to the RyR1 Tetramer
Source: Cells. 2025 Jan 21;14(3):157. doi: 10.3390/cells14030157 (PMC11817637; doi:10.3390/cells14030157)

Complex Actions of FKBP12 on RyR1 Ion Channel Activity Consistent with Negative Co-Operativity in FKBP12 Binding to The RyR1 Tetramer

Spencer J. Richardson [1,†], Chris G. Thekkedam [2,†], Marco G. Casarotto [3], Nicole A. Beard [4] and Angela F. Dulhunty [5,*]

[1] CHEM1—Biotechnology, IP Australia, Woden Valley, ACT 2606, Australia; spencer.sichardson@ipaustralia.gov.au
[2] Developmental and Regeneration Biology Laboratory, Victor Chang Cardiac Research Institute, 405 Liverpool St, Darlinghurst, NSW 2010, Australia; c.thekkedam@victorchang.edu.au
[3] Biomolecular Interactions Group, Research School of Biology, Australian National University, Canberra, ACT 2601, Australia; marco.casarotto@anu.edu.au
[4] Muscle Proteomics Group, Centre for Research in Therapeutic Solutions, University of Canberra, Bruce, ACT 2617, Australia; nicole.beard1971@gmail.com
[5] Muscle Research Group, Eccles Institute of Neuroscience, John Curtin School of Mecical Research, Australian National University, Canberra, ACT 2601, Australia
* Correspondence: angela.dulhunty@anu.edu.au
[†] These authors contributed equally to this work.

Manuscript Figures 1 and 2 are presented on pages 2 and 9 respectively for easy reference.

Supplementary Figures S1 to S5 (from Manuscript Figure 1) are presented on pages 3 to 8.

Supplementary Figures S7 and S8 (from Manuscript Figure 2) are presented on pages 10 and 12.

The following format is used in all of the Supplementary figures.
Top Left – Original Gel, formally annotated, with relevant lanes outlined with red boxes.
Top Right – The relevant panel presented in the manuscript.
Bottom – the original gel with original up at the time of the experiment.

Manuscript Figure 1

Figure 1

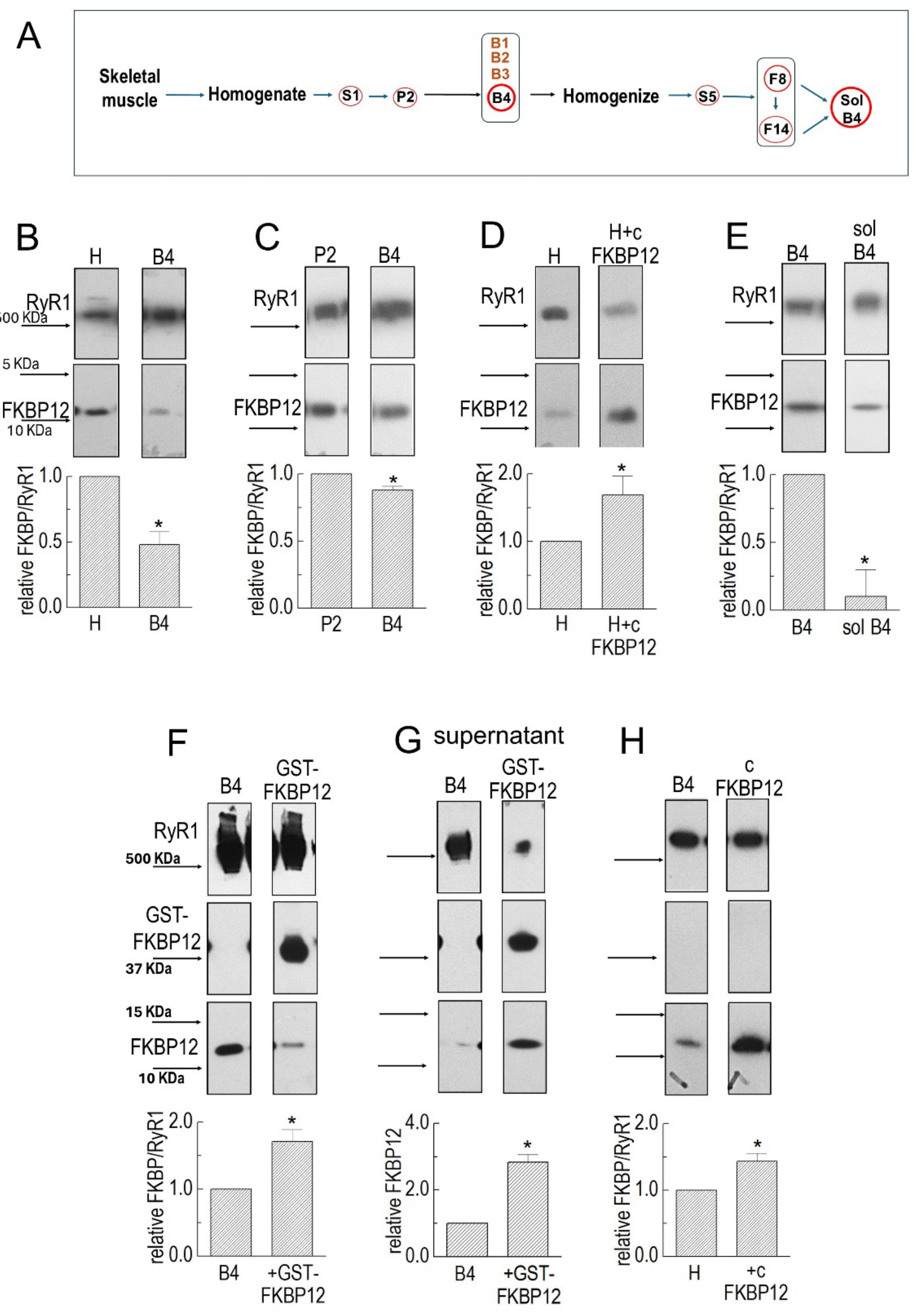

Supplementary Figure S1.

Figure1B

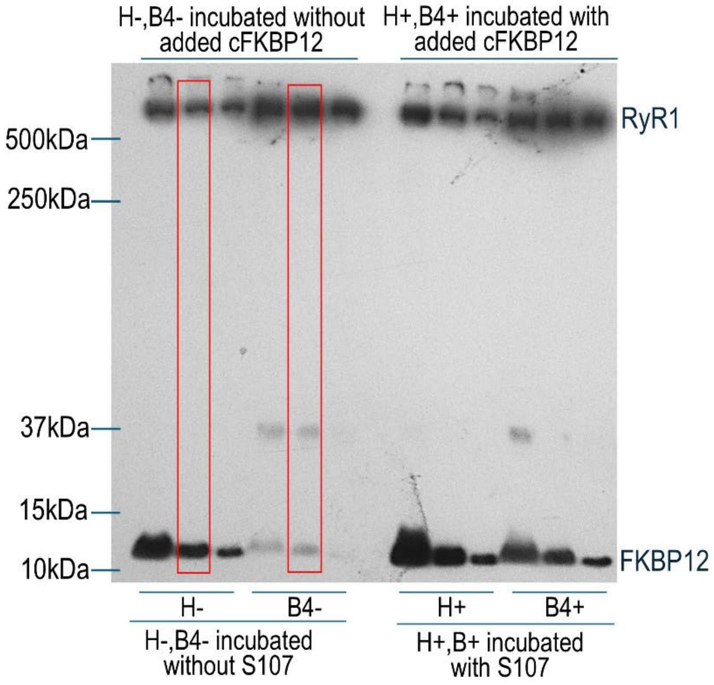

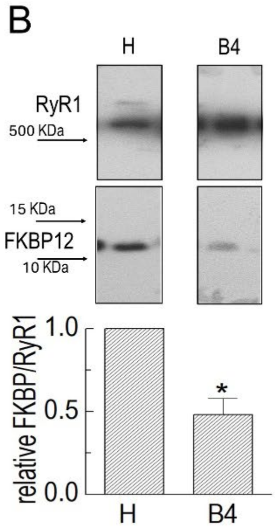

Original photograph of Western Blot

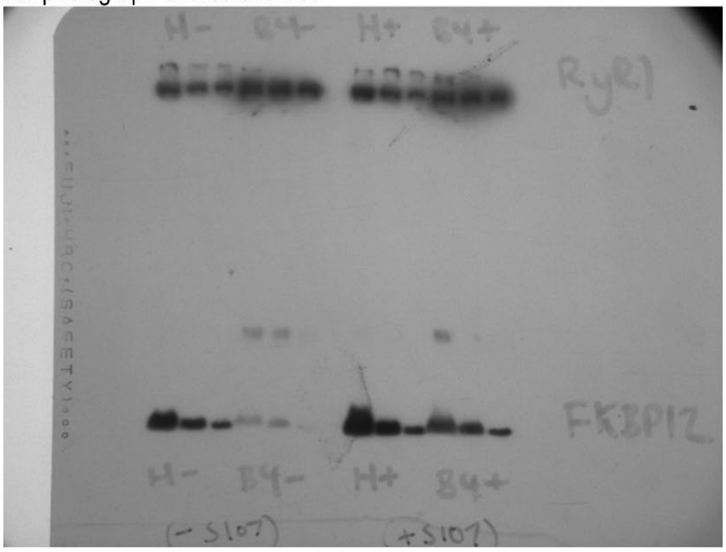

Supplementary Figure S2.

Figure1C

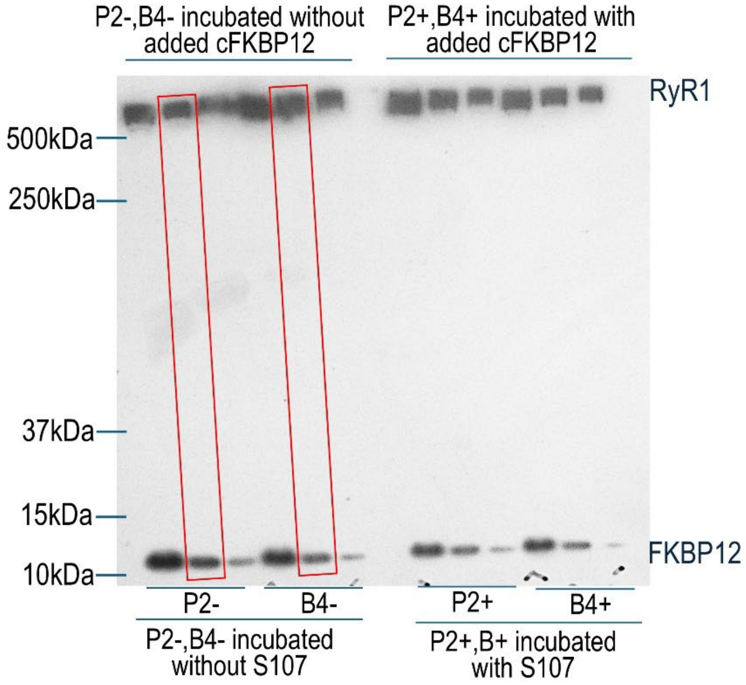

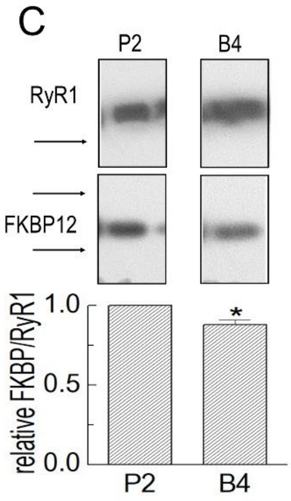

Original photograph of Western Blot

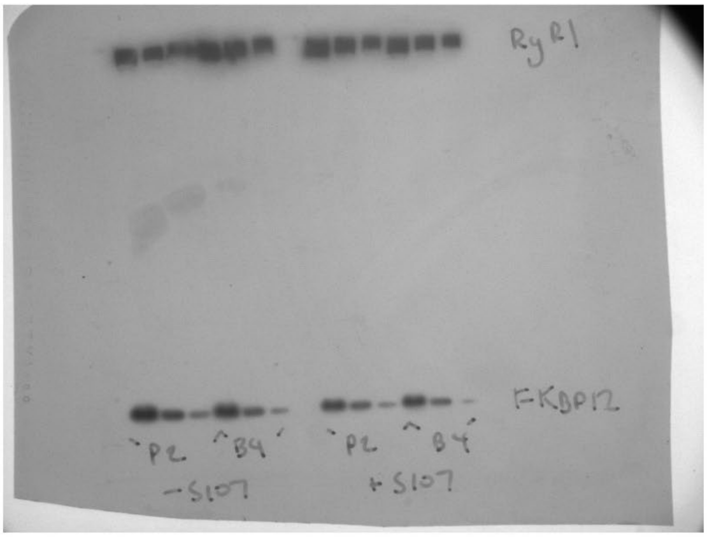

Supplementary Figure S3

Figure1D

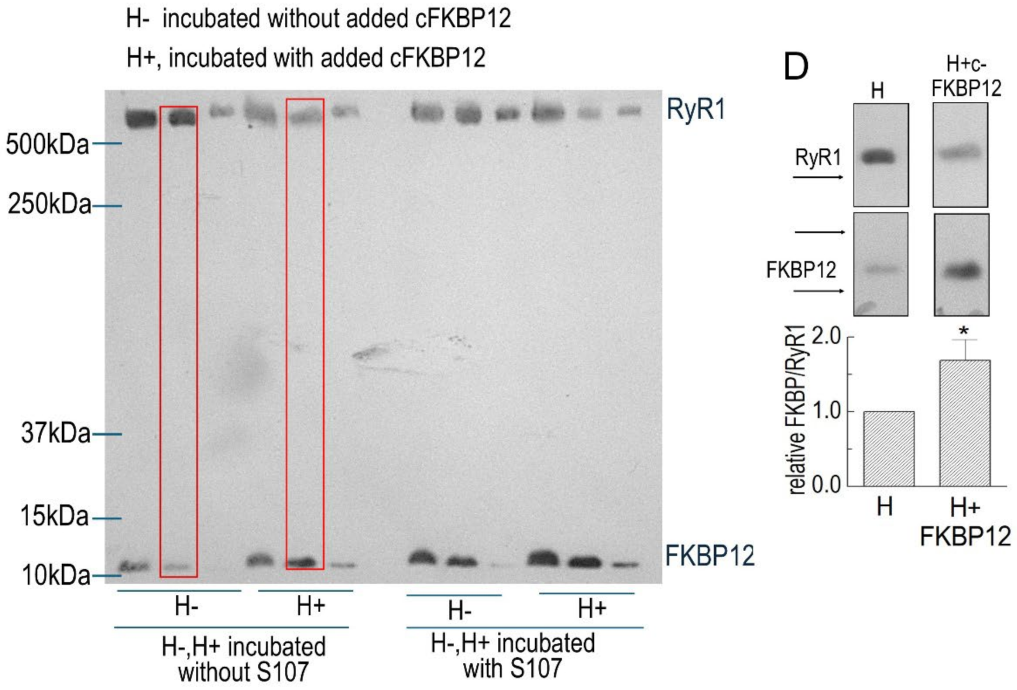

Original photograph of Western Blot

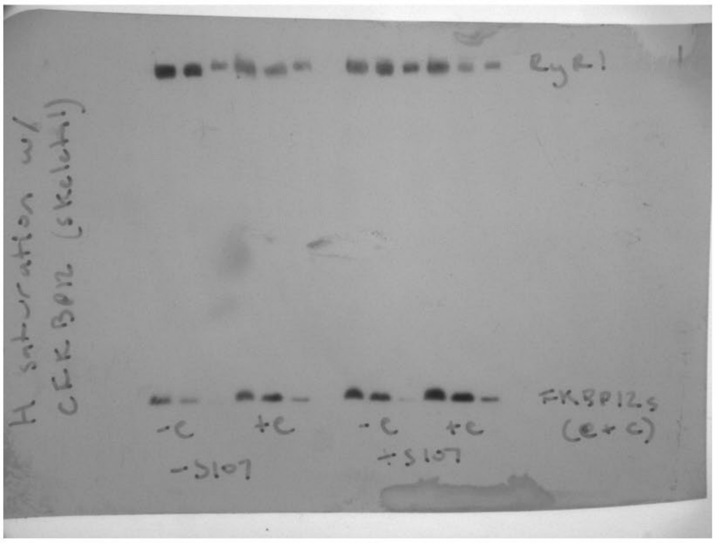

Supplementary Figure S4

Figure1E

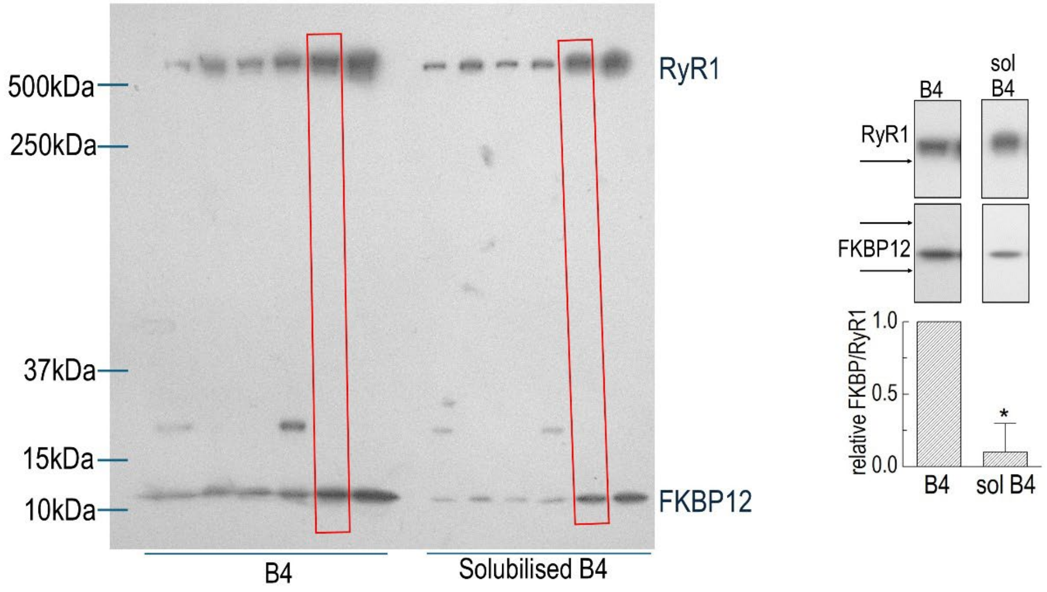

Original photograph of Western Blot

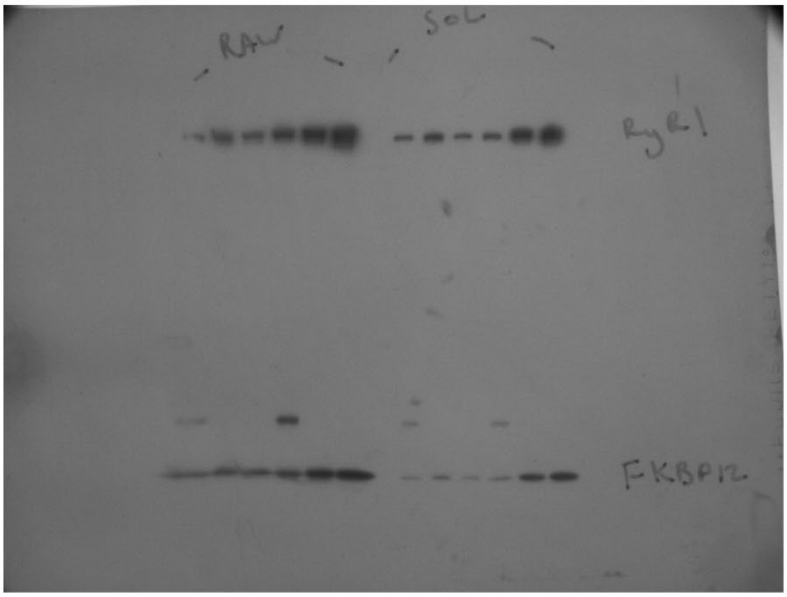

Supplementary Figure S5

Figure1F

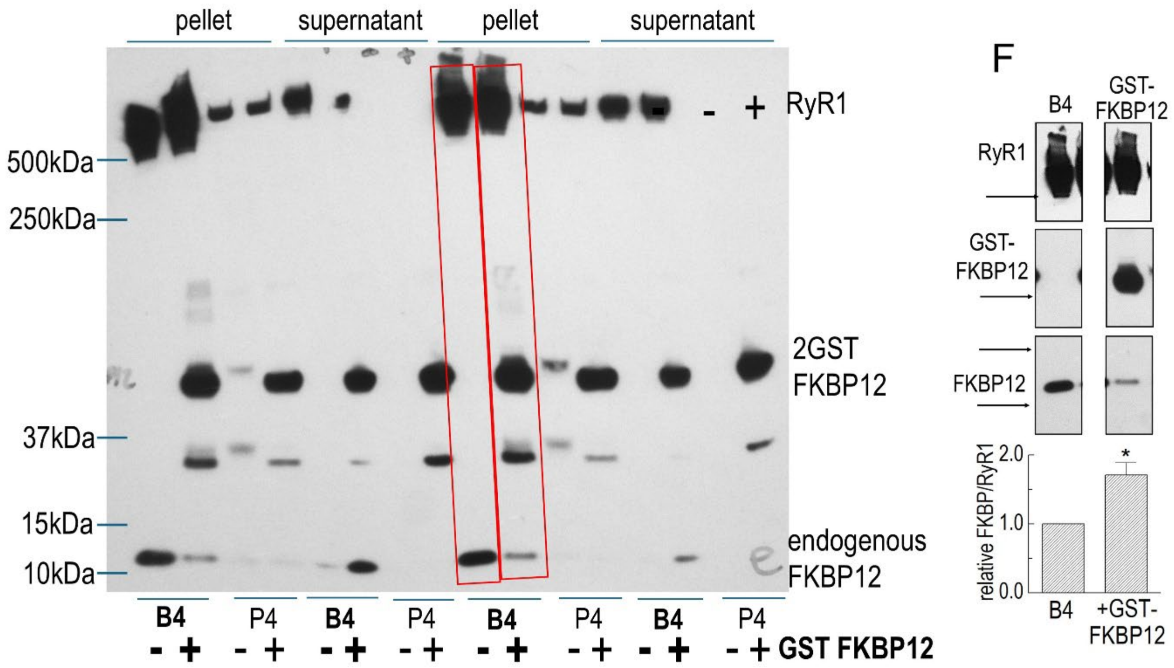

Original photograph of Western Blot

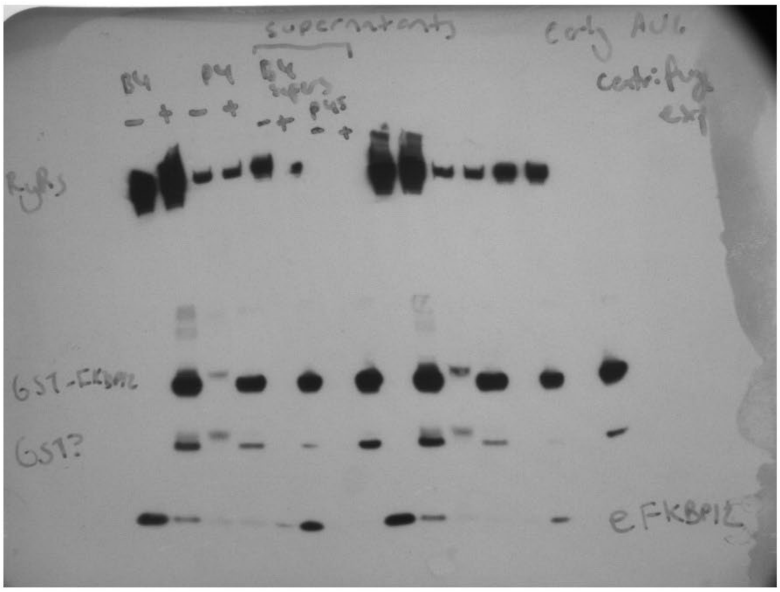

Supplementary Figure S6

Figure1G

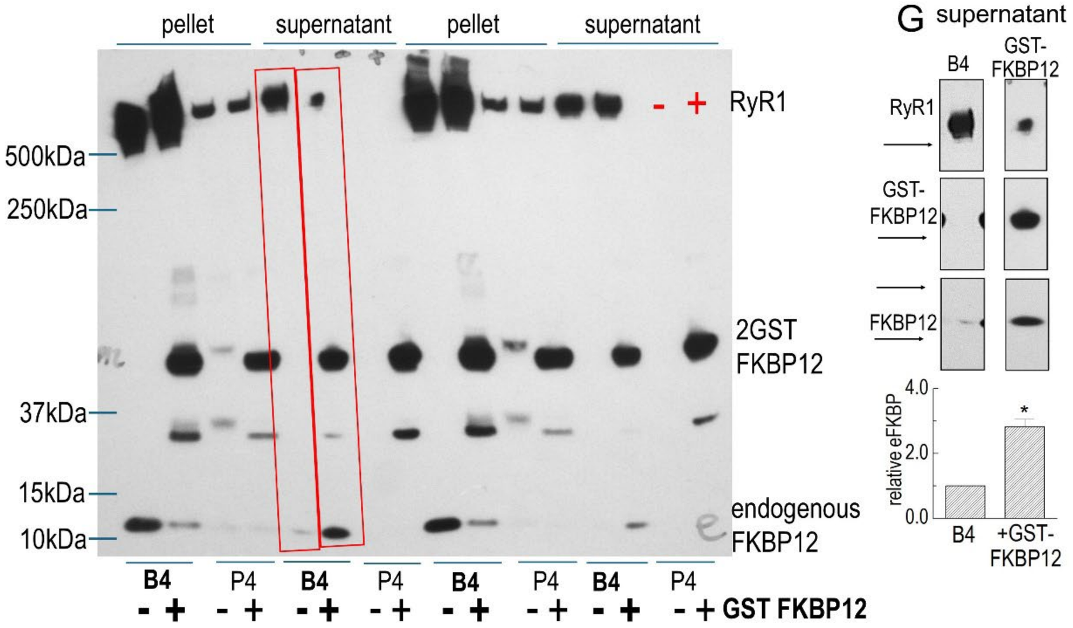

Original photograph of Western Blot

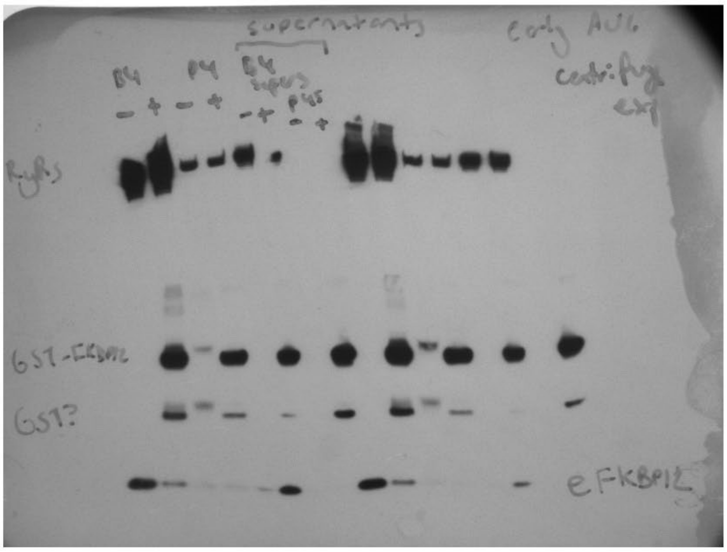

Supplementary Figure S7

Figure1H

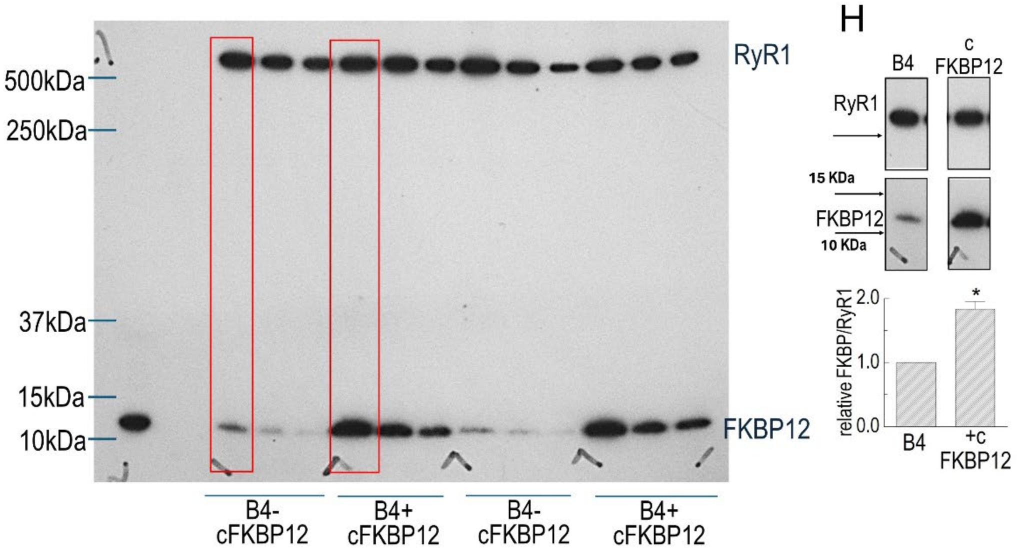

Original photograph of Western Blot

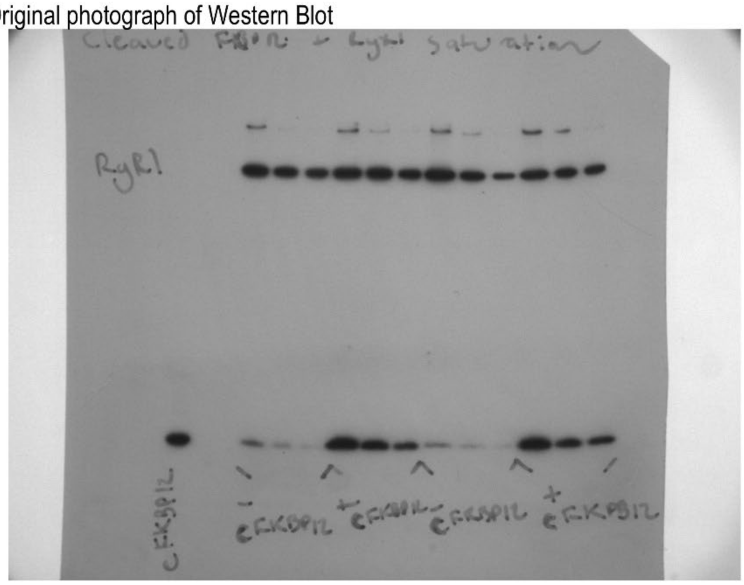

Manuscript Figure 2

Figure 2

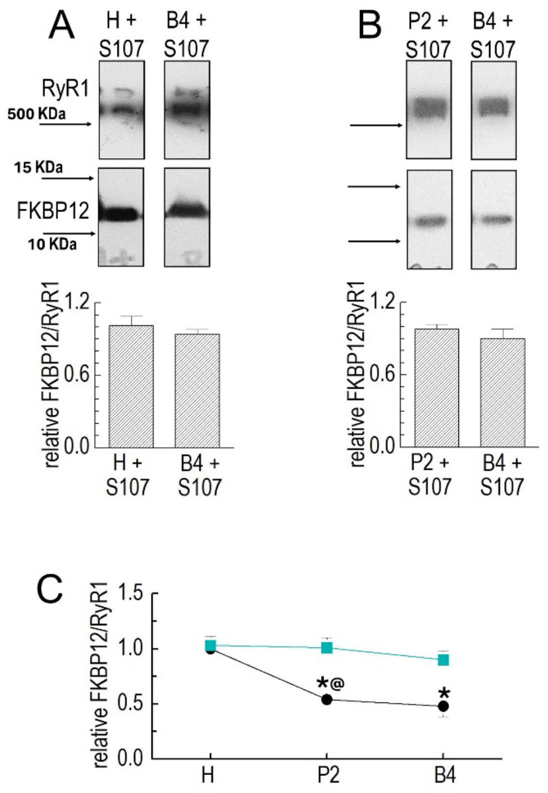

Supplementary Figure S8

Figure 2A

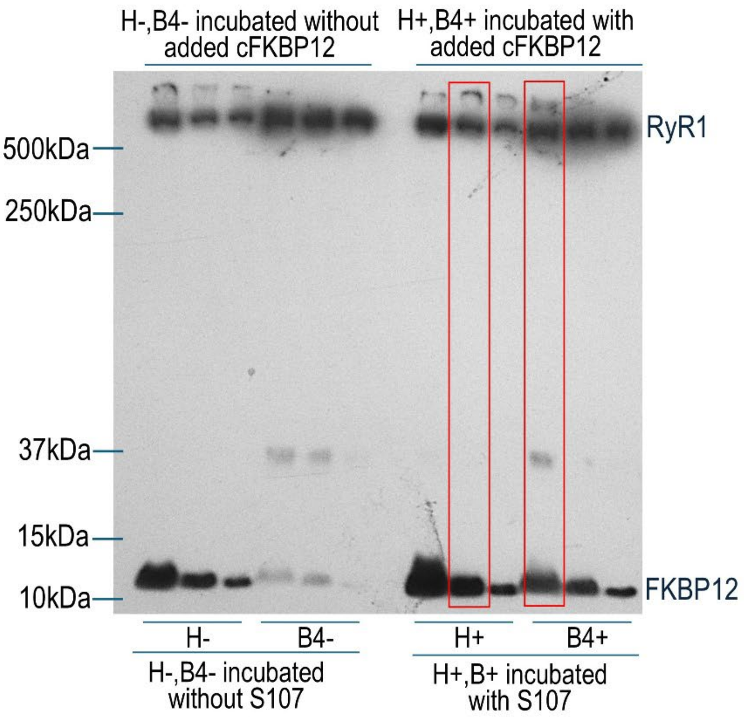

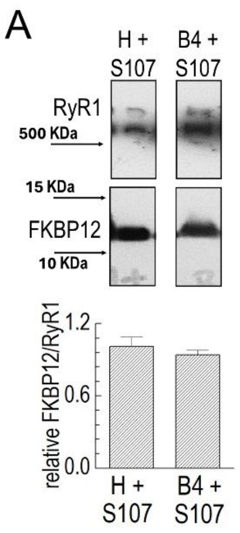

Original photograph of Western Blot

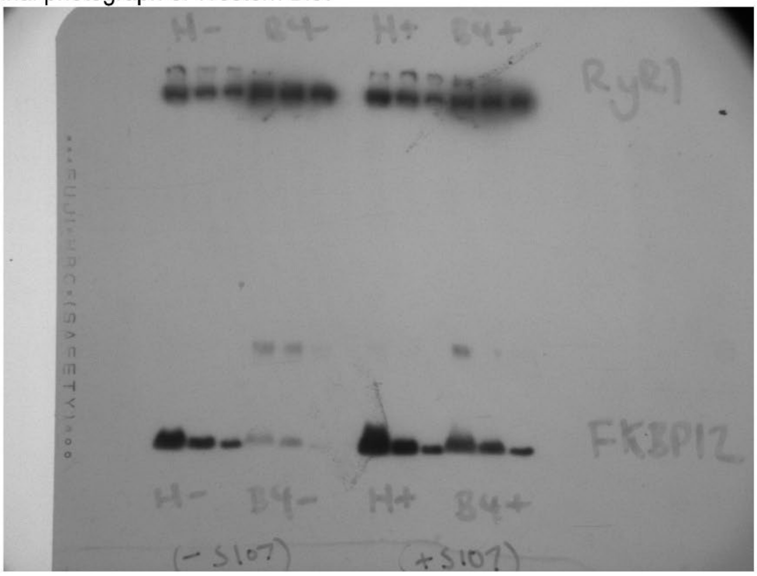

Supplementary Figure S9

Figure2B

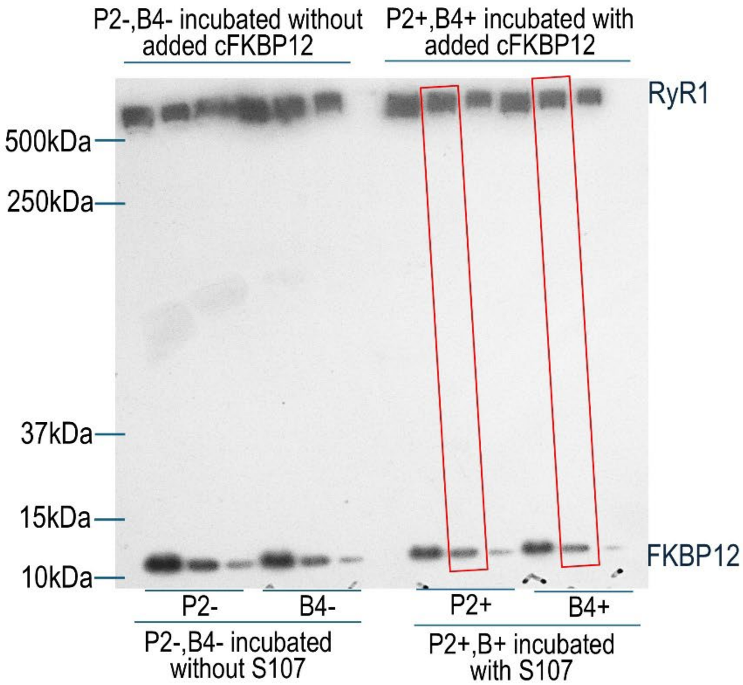

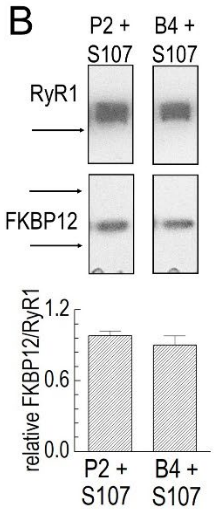

Original photograph of Western Blot

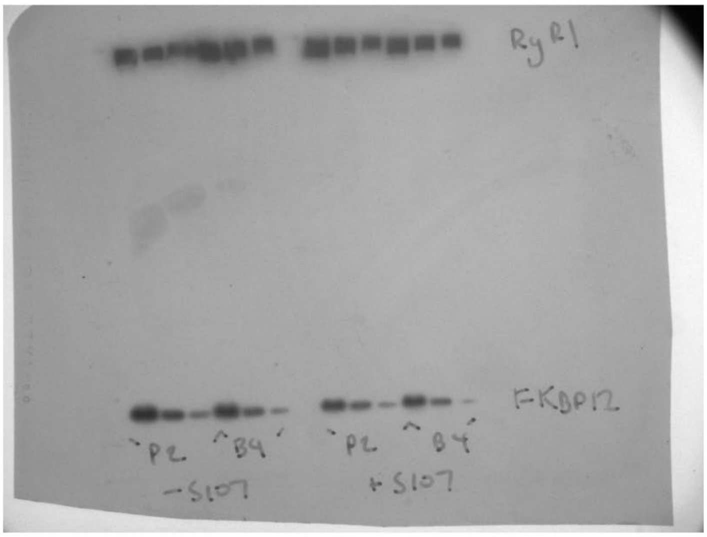

Supplement: Supplementary file 1 [file cells-14-00157-s001.zip › cells-3415379-supplementary-final.pdf]
